# Claudins in Cancer: A Current and Future Therapeutic Target

**DOI:** 10.3390/ijms25094634

**Published:** 2024-04-24

**Authors:** Caroline Hana, Nyein Nyein Thaw Dar, Michael Galo Venegas, Michel Vulfovich

**Affiliations:** Hematology/Oncology Department, Memorial Healthcare System, Pembroke Pines, FL 33028, USA; nthawdar@mhs.net (N.N.T.D.); mgalovenegas@mhs.net (M.G.V.);

**Keywords:** claudin, monoclonal antibodies, carcinoma, targeted therapy, CAR-T cell therapy

## Abstract

Claudins are a family of 27 proteins that have an important role in the formation of tight junctions. They also have an important function in ion exchange, cell mobility, and the epithelial-to-mesenchymal transition, the latter being very important in cancer invasion and metastasis. Therapeutic targeting of claudins has been investigated to improve cancer outcomes. Recent evidence shows improved outcomes when combining monoclonal antibodies against claudin 18.2 with chemotherapy for patients with gastroesophageal junction cancer. Currently, chimeric antigen receptor T-cells targeting claudin 18 are under investigation. In this review, we will discuss the major functions of claudins, their distribution in the normal as well as cancerous tissues, and their effect in cancer metastasis, with a special focus on the therapeutic targeting of claudins to improve cancer outcomes.

## 1. Introduction

The neighboring cells in the epithelial and endothelial sheets are connected by various types of cell–cell junctions, which can be classified as tight junctions (TJs), adherens junctions, and desmosomes [1]. TJs are the main intercellular junctions, which play an important role in the formation of epithelial and endothelial barriers. They have an important role in protecting the internal organs but also form selective permeability barriers that facilitate or restrict the movement of substances among the intercellular spaces. They also play a role in conferring polarity to the epithelial cells [2,3]. TJs can be classified into bicellular TJs (bTJs), between two adjacent cells, and tricellular TJs (tTJs), between three adjacent cells [4]. One of the first molecules described in the structure of bTJs was occludin, which was described as a ~65 kDa protein localized at the TJ of epithelial and endothelial cells, with a hydrophobic NH2 terminal, potentially embedded in the membrane, and a hydrophilic COOH terminal [5]. Knockout of the gene encoding the occludin protein did not result in disruption of the tight junctions. This, subsequently, led to the identification of another set of membrane proteins important for TJ formation. These measured 22 kDa, were composed of four transmembrane domains, and were referred to as claudin 1 and claudin 2 [6]. Another important tTJ protein is tricellulin, also composed of four transmembrane proteins, which is present in higher concentrations at the tTJ and is essential for sealing the tTJ [4].

Claudins (CLDNs) are key proteins that make up the TJ stands. They are integral for the barrier function of the TJ complexes and have a role as pore-forming channels in maintaining the solutes and water permeability in epithelial and endothelial cells [7,8]. In mammals, they are a family of 26 (human) or 27 (rodents) proteins [9,10]. Our knowledge of CLDNs has increased exponentially in recent decades, in which we have discovered their role in cellular projections and cell mobility through the interaction of the CLDNs’ second extracellular domain with the extracellular matrix. This was highlighted via the correlation between CLDN1 expression and the expression of proteins that are important for cell adhesion and actin cytoskeleton remodeling [11]. Additionally, advanced research showed that CLDNs regulate cell signal transduction, proliferation, dedifferentiation, and distant metastasis in cancer biology.

One of the main characteristics of CLDNs is their differential expression among normal tissues as well as their altered expression in cancer tissues, which make them a potential antineoplastic therapeutic target [12,13] Despite what we know about CLDNs, the molecular mechanisms and patterns of CLDNs’ expression in cancers are still being unraveled. Similarly, targeting CLDNs as antineoplastic therapy is being further explored. The goal of this review was to provide an overview of the structure, function, and expression of CLDNs in different tissues, and to provide a deeper insight into their expression in different cancers as well as the current clinical trials targeting CLDNs.

## 2. Tight Junctions and the Structure of the Claudin Proteins

**Figure 1:**
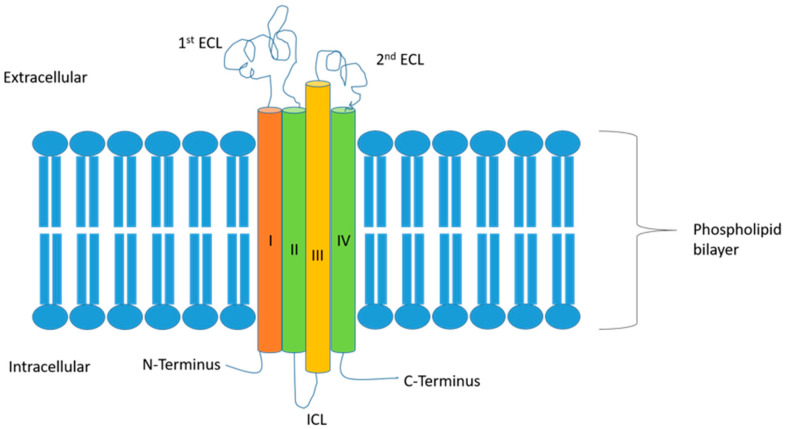
Simplified diagram of the common structure of claudins with 4 transmembrane domains (I through IV) spanning the thickness of the phospholipid bilayer, with 2 extracellular domains, a larger 1st domain and a smaller 2nd domain. ECL: extracellular loop; ICL: intracellular loop.

Overall, CLDNs constitute a highly related family of proteins, which are composed of four transmembrane segments, a large extracellular loop containing a consensus sequence motif, a second shorter extracellular loop, an intracellular loop, an internal C-terminus, and a very short internal N-terminal region [6]. The two extracellular loops are crucial for the formation of the paracellular barriers and electrolytes’ exchange, which, in turn, regulates the permeability characteristics of the TJs (Figure 1). Rajagopal and Nangia examined the structure of CLDN5 and CLDN15. In their work, they investigated the cis and trans conformations of these two CLDNs, showing that in CLDN5, the cis and trans interactions seal the paracellular space, while in CLDN15, they form breaks in the paracellular space, leading to pore formation [14]. This translates to their corresponding functions, where CLDN5 is a barrier-forming protein while CLDN15 is a channel-forming one [14].

CLDNs mostly have similar structures, especially in the membrane-spanning regions. The exceptions to this are CLDN16, 18, and 23. These have extensions at different parts of the CLDN molecule, for example, CLDN16 has a 66-amino-acid extension at the N-terminus. This extension is in the second extracellular loop of CLDN18, while CLDN23 has a longer C-terminal tail. CLDNs 6 and 9 are the most similar, followed by CLDNs 3 and 4, and CLDNs 1 and 7 [9,15].

When examining the half-life of CLDN4, it was found to be as short as 4 hours. This highlights the dynamic function of CLDNs in their barrier and pore formation [16].

## 3. Physiological Functions of Claudins

CLDNs exert different cellular membrane functions including a barrier function, as well as paracellular permeability.

The barrier characteristics of a given tissue and a given CLDN seem to be reliant on the combination of the CLDNs that are expressed and on the manner in which they copolymerize [17]. Some CLDNs exert their function via interaction with other CLDNs, e.g., CLDN23, which functions via heteromeric cis and heterotypic trans interactions with CLDN3 and CLDN4 [18], while others function independently, as in the case of channel-forming CLDNs −2, 10a, 10b, and −15 [19].

On the other hand, CLDNs have a role in charge- and size-selective paracellular permeability [18], for example, CLDNs 2, 7, 10, 15, and 16 increase paracellular cation permeability [20,21], while CLDNs 4, 5, 8, 11, 14, and 19 decrease paracellular cation permeability [22,23,24]. Transepithelial electrical resistance (TER) is one of the easiest and most sensitive measures of barrier strength. As an approximation, epithelia are viewed as an electrical circuit of batteries and resistances. TER is broadly divided into transcellular resistance and paracellular resistance, which, in turn, are the sum of the TJ and the intercellular space [25]. Raya-Sandino et al. demonstrated that in vitro model human intestinal epithelial cells overexpressing CLDN23 displayed a surge in transepithelial electrical resistance. Additionally, knockdown of CLDN23 resulted in decreased resistance values at all time points measured at 5 days of confluency, with a significant increase in Na+ and Cl− permeabilities, proving the role of CLDN23 in the permeability and the barrier function of TJ [18]. Nevertheless, the role of CLDNs in anion exchange has been more controversial, e.g., CLDN 7 knockdown in some studies has been reported to decrease chloride permeation, while, in other studies, its overexpression was linked to the same outcome [26,27].

In addition, CLDNs have a role in cellular projections and cell mobility through the interaction of the CLDNs’ second extracellular domain with the extracellular matrix, as shown in a study of CLDN-4, where knockdown of CLDN4 resulted in decreased cell migration. This was noted in the cultured normal and tumor cells [11,28].

## 4. Claudins’ Expression in Different Tissues

Mammalian CLDNs can be divided into classic and non-classic CLDNs. The classic CLDNs include 1–10, 14, 15, 17, and 19, while the non-classic CLDNs include 11–13, 16, 18, and 20–24. The main structural difference is that non-classic CLDNs have a longer C-terminus [29].

Each CLDN protein has a unique expression pattern based on the cell or tissue type (Table 1), resulting in tissue-specific barrier characteristics [17].

## 5. Claudins’ Expression in Cancer

Our knowledge of CLDNs has greatly expanded over the last few years. They are noted to portray different expressions among different cancers, and many of them correlate with clinicopathological outcomes. Identification of the differential expression of CLDNs in cancer has helped in identifying some of the molecular pathways that can be targeted for treatment. Table 2 summarizes the current knowledge about CLDNs’ expression among different cancers.

## 6. Claudins’ Regulation in Cancer

### 6.1. Genetic Alterations (Amplification)

The expression of CLDNs can be regulated at a transcription or a translation level through multiple mechanisms; for example, CLDN1 expression is inhibited at the mRNA and protein levels by overexpression of the Slug or Snail transcription factors. An inverse correlation in the levels of CLDN1 and Slug transcripts was observed in invasive breast cancer [132]. CLDN1 expression was also suppressed by the transcription of the tumor suppressor factor RUNX3 in gastric cancer [133].

Cho et al. examined the regulation of CLDN7 and showed a role for Ras-related protein 15 (Rab25) in the regulation of CLDN7 via protein stabilization, causing upregulation of CLDN7 and hence reduced colon cancer cell invasiveness [134].

In cervical cancer, CLDN4 was found to be upregulated through the function of Twist1, a helix–loop–helix transcriptional factor. When activated, Twist1 binds to the C:DN4 promoter, causing transactivation of expression [94].

### 6.2. Epigenetic Modifications

DNA methylation has been shown to alter the expression of CLDNs. DNA promoter hypermethylation is associated with downregulation of CLDN1 and CLDN7 in breast cancer and CLDN11 in gastric cancer cells [135].

CLDN1 expression was also shown to be regulated through the modulation of mRNA stability in colon cancer cells in a histone-deacetylase-dependent mechanism [136].

Decreased CLDN2 levels have been reported with the use of azacitidine, a DNA methylation inhibitor, and trichostatin A and sodium butyrate, histone deacetylase inhibitors, giving these chemicals the potential to have an anti-cancer effect [137].

CLDN3 and CLDN4 expression is regulated by DNA methylation and histone deacetylation, as shown by Honda et al., who examined ovarian cancer cells and noted that cells overexpressing CLDN3 and CLDN4 had low DNA methylation and high histone H3 acetylation of the critical promoter region of CLDN4 [138,139]. In ovarian cancer, loss of repressive histone methylations, including H3K27me3 and H4K20me3, is associated with increased expression of CLDN3 and CLDN4 [140]. In urothelial bladder cancer, hypomethylation of the CLDN4 promoter region was associated with cancer metastasis, and so hypermethylation of CLDN4 represents a new potential target of therapy for bladder cancer [141].

Enhancer of zeste 2 (EZH2) is a histone methyltransferase that has a role in epigenetic gene silencing and is aberrantly expressed in colorectal cancer (CRC). Maryan et al. showed that in CRC, EZH2 occupies the CLDN23 gene, resulting in gene silencing [142].

### 6.3. Ligand-Dependent CLDN Regulation

Genetic and epigenetic modifications are not the only mechanisms for regulating CLDNs’ expression in different cancer cells. In breast cancer, CLDN6 expression is driven by ERβ in a ligand-dependent manner. This was explored by investigating the effect of 17β-estradiol on breast cancer cells. It was noted that it enhances the expression of Erβ, but not ERα. On the other hand, after knocking down ERβ, CLDN6 expression was not enhanced with treatment by 17β-estradiol. 17β-estradiol treatment was also noted to significantly upregulate the mRNA and protein expression of CLDN6 in a dose-dependent manner [100]. Interestingly, on the other hand, the CLDN6 effect was modulated by ERα in endometrial carcinoma [102].

## 7. Role of Claudins in Cancer

### 7.1. Tumor Suppressor Effect

TJ proteins are believed to work as tumor suppressors because they are the hallmark of epithelial cells and their expression decreases in parallel with the progression of cancer [143].

Genetic analysis of human breast tumor cells showed that a significant number of TNBC tumors had a low expression of CLDN genes (i.e., CLDN3, −4, −7, and E-cadherin), which gives a poor prognosis [144]. In a further study, in vivo mice models demonstrated that activation of the RAS system in the luminal epithelial cells could be the origin of the development of basal-like, CLDN-low mammary breast cancer [145].

CLDN7 is also reported as a tumor suppressor protein in colorectal cancer. A CLDN7 knockout mice model and colorectal cell lines showed significant tumor growth, tumor cell migration, and inhibition in apoptosis via the SOX-9-mediated Wnt/β-catenin signaling pathway [134,146]. Moreover, CLDN7 was shown to have a similar tumor-inhibitory effect in oral squamous cell carcinoma [147].

Another in vitro study investigated the role of CLDN17 in head and neck cancer cells, where CLDN17 gene expression profiles in oral cancer tissues were analyzed. There was an association of lower CLDN17 expression with higher tumor staging, poorer tumor histological grading, and worse clinical prognosis [119]. Therefore, CLDN17 was proposed to have a tumor suppressor effect in oral cancer by inhibiting epithelial–mesenchymal transformation, tumor invasion, and migration.

### 7.2. Tumor Promoter Effect

Although CLDNs are believed to function as tumor suppressors due to their sealing effect at TJs, they are also found to have oncogenic properties such as promoting cell growth, proliferation, invasion, migration, and metastasis.

CLDN1 promotes oral squamous carcinoma cell invasion by activating membrane-type matrix metalloproteinase (MT1-MMP) and matrix metalloproteinase-2 (MMP-2) [148]. In human CRC, there is an increase in CLDN 2 expression, which promotes the self-proliferation of CRC cells, indicating a potential role in the pathogenesis of this type of cancer [7,149]. This was re-demonstrated in colorectal cancer, where CLDN2 was upregulated and associated with a poor prognosis. Wei et al. demonstrated that CLDN2 suppression promoted N-myc downstream regulated gene 1 (NDRG1) transcription, which prohibited tumor progression and metastasis in vitro and for in vivo models [150].

Similarly, a study by Agarwal et al. showed that CLDNs 3 and 4 were highly expressed in ovarian cancer cell lines, which increased cell survival and promoted cancer metastasis by enhancing MMP-2 activity [151]. CLDN4 is also a tumor promoter gene in urothelial bladder cancer [141]. Likewise, in breast cancer, cell proliferation, migration, and tumor growth are enhanced via CLDN4-adhesion signaling, which works through interacting with LXRβ by targeting the AKT phosphorylation site S432 in LXRβ [87].

CLDN6 is an oncofetal antigen that is typically silent in normal tissues, but reactivated in germline tumors like testicular, ovarian, and uterine cancer. This implies or suggests that CLDN6 may have potential as a diagnostic marker or even a therapeutic target in these cancer types [7]. A similar tumor promoter effect of CLDN6 was demonstrated in human hepatocellular carcinoma (hHCC). High expression of CLDN6 was associated with tumor differentiation of hHCC according to the Cancer Genome Atlas (TCGA) database. In an in vitro study, silencing the CLDN6 gene resulted in decreased tumor proliferation, migration, and invasion with upregulated E-cadherin and downregulated N-cadherin and vimentin [152].

In vitro studies in bile duct carcinoma showed that blocking CLDN18 via antibodies could reduce cell proliferation. The tumor promoter effects of CLDN18 went through epidermal growth factor, RAS, and extracellular-signal-related kinase (ERK) 1/2 pathways [153]. Likewise, Zhou et al. demonstrated that CLDN18 regulates cancer stem cells in lung cancer [154].

### 7.3. Role in Tumor Microenvironment

TNBCs that are claudin-low tumors have an enrichment of tumor-associated macrophages (TAMs). They have a spindle-like morphology and are known for their highly mesenchymal nature. They are associated with a higher incidence of TAM and worse overall survival. In normal and cancerous tissues, TAM survival, proliferation, and differentiation are promoted by the downstream effects of the interaction of colony stimulating factor 1 receptor (CSF1R), a receptor tyrosine kinase, with CSF1 and IL34 [155].

Singh et al. showed that the use of chemotherapy, namely low-dose cyclophosphamide coupled with the pharmacologic inhibition of TAMs using either a small-molecule CSF1R inhibitor or an anti-CSF1R antibody, resulted in an expansion of CD8+/CD4+ T cells and B cells in these TNBCs in the treated mice, as well as polyclonal expansion T cells that exhibited memory cell phenotypes. This also resulted in a significant response in these treated mice [156]. In CRC, loss of expression of CLDN1 is significantly associated with NF-κB activation (*p* < 0.001), high SNAI (*p* < 0.001), and low E-cadherin (*p* < 0.001). It is also noted with low CD3- and CD8-positive lymphocytes [65].

Gao et al. studied the correlation of CLDNs and the tumor immune microenvironment in ovarian cancer. They noted that most gene markers of dendritic cells, monocytes, M1 macrophages, TAMs, and NK cells negatively correlated with CLDN6 expression but positively correlated with CLDN10 expression. These findings indicated that CLDNs may play an important role in immunotherapy in the future [74].

### 7.4. Tumor Markers

Transmembrane types of CLDN proteins are highly expressed in precancerous and cancerous cells, and they have shown potential as tumor markers.

In an immunohistochemistry (IHC) study of CLDN4 in pleural and peritoneal fluid or tissue biopsies, the CLDN4 stain was strongly positive in primary carcinoma and metastatic lesions but not in mesothelioma [157]. Hence, CLDN4 can be used as a tumor marker to differentiate neoplastic metastases from mesothelioma. The sensitivity and specificity of CLDN4 were evaluated by IHC in cell blocks, which included non-conclusive encompassing atypia of undetermined significance, suspicious for malignancy, and benign cases. Interestingly, CLDN4 was positive for 100% of adenocarcinoma cases and negative for 100% of mesothelial and mesothelioma effusions. Overall, the sensitivity, specificity, positive predictive, and negative predictive values for CLDN4 in metastatic adenocarcinoma were 85%, 100%, 100%, and 75%, respectively [158]. Similar results were noted by Elhosainy et al., where CLDN4 exhibited 95.8% sensitivity and 96.9% specificity in the detection of metastatic adenocarcinoma [159].

Similarly, studies of lung biopsy tissue showed that adenocarcinoma tumors had the highest staining of CLDN4 and atypical adenomatous hyperplasia cells had higher scores compared to the normal alveolar epithelium, which also indicated that CLDN4 is involved in the early tumorigenesis process [160]. Another important novel tumor marker for malignant pleural mesotheliomas is CLDN15 [161].

CLDN6 is known as a marker for pluripotent stem cells because it is highly expressed in undifferentiated cells but not in normal tissue [162]. Despite having almost no expression in normal adult tissue, CLDN6 is expressed at elevated levels in multiple human cancers including ovarian and endometrial malignancies. This differential expression makes CLDN6 an ideal target for the development of a potential therapeutic antibody–drug conjugate (ADC) [163].

In gastrointestinal cancer, membrane-bound CLDN7 and CLDN18 were proven to offer reliable immunohistochemical markers for the diagnosis of pancreatic ductal neoplasia [164], and CLDN18 has high sensitivity and specificity for diagnosing biliary tract adenocarcinoma or intraepithelial neoplasia [165]. CLDNs 1, 3, and 7 and are highly expressed in colorectal adenocarcinoma, and CLDN4 staining results were strong in colorectal and pancreatic cancer tissue samples [166].

### 7.5. Role of Claudins in Cancer Metastasis

CLDNs also have a role in the epithelial-to-mesenchymal transition (EMT), one of the most important functions of CLDN proteins in disease progression [24].

According to the EMT hypothesis, the epithelial cell transforms to a mesenchymal cell by losing its epithelial cell marker (e.g., E-cadherin) and gaining a mesenchymal cell marker (e.g., N-cadherin), which allows epithelial cells to acquire mesenchymal characteristics such as an increased migration rate. This phenomenon is one of the established phenomena of cancer progression [167].

In colon cancer, nuclear localization of CLDN1 was noted frequently, and manipulation of CLDN1 expression significantly affected the EMT marker changes and distant metastasis during in vitro and in vivo studies [29,168]. For example, in murine models of metastatic CRC, CLDN1 is a direct downstream target and effector of lin-28 homolog B (LIN28B), an RNA-binding protein that directly binds to and post-transcriptionally regulates CLDN1 mRNA. LIN28B-mediated CLDN1 expression enhances collective invasion, cell migration, and metastatic liver tumor formation [169]. The importance of CLDN1 in cell migration was also illustrated by Tu et al. using the FH535 molecule in CRC cells, which inhibits the Wnt signaling pathway. This resulted in lower levels of CLDN1, induction of G2/M arrest, and inhibition of cell proliferation and migration [170]. Likewise, overexpression of CLDN23 in CRC cells was associated with decreased cell adhesion and increased cell proliferation and migration ability [130]. In TNBC, CLDN1 correlates with the expression of β-catenin, which is an important oncogene and a major contributor to the EMT [69].

Also, CLDN1 expression in hepatocellular carcinoma (HCC) promotes the EMT via the c-Abl/Raf/Ras/ERK signaling pathway [29,171]. Therefore, CLDN-1-targeted therapy may offer a novel antineoplastic therapy in the future. In a study of invasive liver fluke *Opisthorchis viverrini*-associated cholangiocarcinoma, a truncated del p53 variant, del p53M213, exhibited gain-of-function. This was associated with a decrease in CLDN1 expression. It was also noted to lack anti-growth functions and instead enhanced migration and invasiveness [172].

Furthermore, an in vitro study of melanoma cells showed that cytoplasmic CLDN-1 promoted metastatic ability and could be blocked by regulating the phosphorylation pathway via protein kinase activity [173]. A similar finding was reported in the follicular thyroid carcinoma cell lines, in which tumor invasion and migration were promoted by CLDN-1 localized in the nucleus [174].

On the other hand, some claudins have an inhibitory effect on the EMT in certain types of malignancies. CLDN1 was shown to have an inhibitory effect on cancer metastasis in lung adenocarcinoma, as the upregulation of CLDN1 suppressed the ERK1/2 signaling pathway. Moreover, CLDN1 also enhances the efficacy of chemotherapy, so CLDN1 is not only a potential prognostic marker but also a predictive marker for chemotherapy benefits in metastatic cancer [175]. CLDN3 inhibits EMT in HCC [176] and lung cancer [177]. In vitro and in vivo studies of pancreatic cancer showed that overexpression of CLDN4 enhanced cell-to-cell adhesion and prevented cancer cells from engaging in invasion and distant metastasis via the transforming growth factor beta (TGF-β) and Ras/Raf/extracellular-signal-regulated kinase pathways [167]. Moreover, CLDN6 was found to have an inhibitory function in breast cancer metastasis by upregulating WIP expression (WIP regulates the actin cytoskeleton autophagy pathways) during in vivo and vitro studies. This finding could explain why low levels of CLDN6 expression were found in metastatic breast cancer [101]. In colon cancer, CLDN6 activates the TYK2/STAT3 pathway, which might suppress the migration and invasion abilities of colon cancer cells [104].

Another great example of how CLDNs’ effects are variable in different cancers is how CLDN6 promotes the EMT in gastric cancer [178] while its downregulation in breast cancer promotes cancer invasiveness and progression [179].

### 7.6. Claudins and Chemoresistant Tumors

CLDNs have overall EMT, tumor invasion, and tumor stemness capacities, which are fundamental factors for developing chemoresistance in cancer. Several mechanisms have been postulated as theories for such chemoresistance.

CLDN1 was demonstrated to cause chemoresistance in CRC via upregulating ephrin type-A receptor 2 (EPHA2) tyrosine kinase, which enhances downstream the AKT signaling pathway, and CD44 expression, which promotes cancer stemness and chemoresistance [62]. This causes 5-FU resistance in colon cancer cell lines [180], cisplatin resistance [181] or doxorubicin resistance [67] in lung cancer, and drug resistance in liver cancer. These findings suggest that CLDN1 is not only a potential prognostic marker but also a predictive marker for chemotherapy benefits in metastatic cancer [175].

Similarly, the suppression of CLDN3 in non-small-cell lung cancer decreased cancer stemness and improved chemosensitivity [182]. CLDN3 and CLDN4 also regulate cisplatin sensitivity in ovarian cancer cells via copper transporter (CTR1), while CLDN7 enhances cisplatin sensitivity in lung cancer cells via the caspase pathway [183].

In ovarian cancer, CLDN4 overexpression was associated with a dampened PARP-inhibitor-mediated antiproliferation response, while inhibition of CLDN4 sensitized the tumor sections to it [184]. In the TNBC cell line, CLDN6 promotes adriamycin-resistant cancer clones via the afadin (AF-6)/ERK pathway [185]. Therefore, CLDNs induce not only tumorigenesis but also treatment resistance in cancer cells.

## 8. Investigational Role of CLDNs in the Early Detection of Cancer

In a study of radiolabeled anti-CLDN4 monoclonal antibodies, when antibodies labeled with ^125^I were injected into mice with severe combined immunodeficiency (SCID) bearing PANC-1 xenografts, the highest uptake was noted in the liver (4.5%) followed by PANC-1 tumors (4%) and the spleen (3.5%). Similarly, increased uptake primarily in the tumor was seen in SCID mice bearing Colo357 cell xenograft tumors (originating from pancreatic carcinoma). Using SPECT-CT, the uptake could be measured, and this revealed that the tumor uptake was 2.5 times that of the spleen and 2 times that of the liver. The investigators concluded that ^125^I-labeled anti-CLDN4 antibody can be used for SPECT-CT to detect pancreatic cancer; however, due to the low-energy gamma photon emission, it can only be used for imaging of small animals, with a sensitive gamma camera [186].

On the other hand, a meta-analysis of the use of CLDN-3 for the evaluation of prostate cancer showed that CLDN3 is indeed one of the strongest two markers overexpressed in cancer when compared with prostate-specific antigen. However, it was deemed not superior to PSMA scanning because its expression was not drastically different between normal and cancerous tissues [187].

Further studies are needed to improve and support the role of CLDNs in cancer diagnosis.

## 9. Therapeutic Targeting of Claudins

Ideally, an effective cancer therapy molecule should meet two criteria: first, restricted expression in specific tissues to avoid side effects, and second, positive expression with exposed epitopes in cancerous tissues for targeted treatment. CLDNs have been identified as meeting both criteria, which make them promising targets for cancer therapy [188].

CLDNs, typically located within the TJ complex in normal tissues, are known to become more accessible in malignant tissues due to extra-junctional mis-localization. This is a unique expression profile that makes CLDNs theoretically attractive targets for selective drug delivery with minimal adverse effects. Several approaches, such as Clostridium perfringens enterotoxins (CPEs), monoclonal antibodies (mAbs), CPE-binding domains (C-CPEs), mAb–drug conjugates, bispecific T-cell engagers (BiTEs), and chimeric antigen receptor (CAR)-T cells, continue to be explored for targeting CLDNs in cancer patients. Ongoing phase I to phase III clinical studies indicate the potential significance of CLDN-targeted agents [188].

Table 3 summarizes the current clinical trials targeting CLDNs.

### 9.1. Monoclonal Antibodies (mAbs)

In 2004, Offner et al. started exploring CLDNs as targets for antibody-based cancer therapies using chicken bodies against extracellular domains of CLDNs 1, 3, and 4 [197]. Suzuki et al. succeeded in generating a mAb (KM3900 (IgG2a)) that targets CLDN4. KM3900 was found to bind to CLDN4 on pancreatic and ovarian cancer cells but not normal cells, causing dose-dependent, antibody-dependent cellular cytotoxicity (ADCC) and complement-dependent cytotoxicity (CDC) in vitro, as well as in vivo tumor growth inhibition in mice models [198].

Zolbetuximab (IMAB362, claudiximab) is a chimeric IgG1 antibody, highly specific to CLDN18.2. Its binding to CLDN18.2 also induces ADCC and CDC. When combined with chemotherapy, zolbetuximab enhances T-cell infiltration and induces pro-inflammatory cytokines [199]. Its safety was investigated in a phase I/II trial [200] (Table 2).

Zolbetuximab combined with interleukin-2 and zoledronic acid was investigated in patients with gastroesophageal junction (GEJ) cancer who failed multiple lines of therapy, and 11 out of 20 patients had disease control [201].

In the SPOTLIGHT randomized, double-blind, placebo-controlled, phase III trial, zolbetuximab combined with mFOLFOX6 was investigated as a first-line treatment for HER2-negative, CLDN18.2-positive, locally advanced unresectable or metastatic GEJ adenocarcinoma. The study showed a significantly improved overall survival of 18.23 months compared to 15.53 months. The results of SPOTLIGHT are promising, as they support zolbetuximab-based therapy for patients with high expression of the CLDN18.2 biomarker. The most common treatment adverse events were nausea, vomiting, and decreased appetite, which are consistent with previous phase 1 and phase 2 studies. This is encouraging as there are no new safety warnings to take into consideration. In sum, this study demonstrated clinically significant benefits for patients with CLDN 18.2-positive, HER-2-negative disease and most likely zolbetuximab is going to be considered as a first-line treatment option in combination with chemotherapy [193].

Monoclonal antibodies targeting CLDNs, particularly CLDN1 and CLDN4, hold promise as therapeutic agents in the treatment of various cancers, with potential synergistic effects when combined with known anti-cancer agents like 5-fluorouracil and anti-EGFR antibodies. For example, there is PDS0330, developed by Fatima et al., which inhibits CLDN-1-dependent CRC progression [81]. Additionally, antibody–drug conjugates have shown promise in inhibiting tumor growth and metastasis in specific cancer types, for example, pancreatic and gastric tumors [188].

### 9.2. Clostridium Perfringens Enterotoxin (CPE)

Clostridium perfringens enterotoxin (CPE) and its C-terminus domain recognize specific amino acid sequences in the extracellular loops of CLDNs 4 and 3. This recognition leads to the disruption of tight junctions and perforation of the plasma membrane, which ultimately causes cell death. This cytotoxic effect has been observed in various cancer types, for example, non-small-cell lung, prostate, gastric, and ovarian cancer. The impairment of tight junctions by CPE disrupts the tumor microenvironment barrier, and this enhances drug delivery to cancer cells, making them more susceptible to anti-cancer drugs and even suppressing metastasis. Conjugation of CPE and anti-cancer drugs produces a carrier for targeted delivery to cancer cells expressing CLDN4. However, the clinical use of CPE may be limited by immunogenicity and potential toxicity, similar to how Clostridium perfringens causes mucosal epithelial damage, food poisoning, and even CPE-induced shock [202].

### 9.3. Chimeric Antigen Receptor T (CAR-T) Cell Therapy

CAR-T cell therapy has been effective in treating B-cell malignancies but faces challenges in solid tumors. Although it has shown promise in targeting CLDNs expressed on solid tumor cells, as demonstrated in preclinical models administered engineered CAR-T cells with high specificity for CLDN6 and CLDN18.2 [203]. For example, the development of CARvac, an RNA vaccine that enhances CAR-T cell engraftment, is a novel strategy to improve the effectiveness of CAR-T therapy. Another alternative is BiTES, a method to target CLDN18.2, potentially enhancing the immune response against cancer cells. Ongoing phase I clinical trials recruiting patients with advanced tumors positive for CLDN18.2 suggest active research and a promising avenue for treating solid tumors [7,188].

Currently, phase I clinical trial are underway to investigate CLDN18.2-targeted CAR-T in patients with unresectable, locally advanced, or metastatic gastric, GEJ, esophageal, or pancreatic adenocarcinoma (NCT05539430) [204].

### 9.4. Calcium and Vitamin D Supplementation

In a large, multicenter, randomized, placebo-controlled, partial 2 × 2 factorial, chemoprevention clinical trial, testing the efficacy of calcium and vitamin D supplementation on rectal adenocarcinoma recurrence, after supplementation for 3–5 years, patients with removed colorectal adenomas did not have a significantly lower risk of rectal adenomas [205]. Despite those findings, subjects from that study were selected to participate in an adjunct trial, where they were randomized to four treatment groups: 1200 mg/d calcium supplementation, 1000 IU/d vitamin D3 supplementation, a combination of both, and aplacebo. Then, biopsies from the normal mucosa were collected at baseline and year 1 of follow-up to examine the expression of the tight junctions’ proteins. It was noted that CLDN1, occludin, and mucin-12 expression increased by 14% (*p* = 0.17), 23% (*p* = 0.11), and 22% (*p* = 0.07) in the calcium group compared to the no calcium group [206]. This finding raises the question about the clinical potential for calcium and vitamin D supplementation in colorectal carcinogenesis and metastasis.

### 9.5. Preclinical Studies

In a study of a cohort of 5FU-resistant CRC cells, the genetic silencing of CLDN1 increased the sensitivity of these cells to 5-FU and inhibited its metastatic potential by regulating the expression of EMT-related genes. Reduced expression of CLDN1 was achieved through the co-treatment of CRC chemoresistant cells with Lactobacillus plantarum-derived metabolites and 5-FU [207].

Similarly, solasodine, an active ingredient isolated from *Solanum nigrum* L., was found to regulate the expression of CLDN2, and it regulated the EMT by modulating the AMPK/STAT3/NF-κB/CLDN2 signaling pathway [208].

Furthermore, decreasing the level of CLDN3 and CLDN4 proteins through knockdown of CLDN3 and CLDN4 in prostate cancer resulted in a 30–40% decrease in prostate cancer cell growth, a 60–65% reduction in cell viability, and cell migration [209].

Plasmacytoma variant translocation 1 (PVT1) is a non-coding RNA transcribed from a gene located in the 8q24 chromosomal region, which has been implicated in multiple cancers. Targeting PVT1 exon 9 expression in a claudin-low breast cancer model via siRNA resulted in re-expression of CLDN4 and a significant reduction in migration [210].

CLDN4 has also been investigated as a target to reduce chemoresistance. In a trial of 4D3, an anti-claudin (CLDN)-4 extracellular domain antibody enhanced paclitaxel-induced growth suppression in TNBC and increased the intracellular paclitaxel concentration and apoptosis [211].

Likewise, CLDN6 has become an interesting target for cancer therapy, and CLDN-6-targeting chimeric antigen receptor (CAR)-T cell therapy has shown positive outcomes in vitro and in mice models [212,213].

## 10. Conclusions

CLDNs, pivotal proteins within the tight junctions, play a crucial role in maintaining epithelial cell polarity and forming permeability barriers. Their expression varies across tissues, contributing to diverse functions and exhibiting distinctive patterns in cancerous tissues. Given their unique expression profiles, particularly with exposed epitopes in malignant cells, CLDNs have emerged as promising targets for cancer therapy.

One of the most extensively studied targets is CLDN18.2, particularly in gastric and gastroesophageal cancer. Phase III clinical trials such as SPOTLIGHT and GLOW have investigated the efficacy of zolbetuximab, a monoclonal antibody, in locally advanced and metastatic gastric and GEJ adenocarcinomas. Results from these trials reveal a modest yet significant improvement in PFS and OS, underscoring its potential as a therapeutic option. Nevertheless, the challenge lies in identifying the patients that will benefit the most from such targeted therapies, raising questions about the feasibility of utilizing CLDN18.2 expression as a predictive biomarker. Additional therapeutic avenues, including CAR-T cellular therapy targeting CLDN18.2, are also under exploration.

Similarly, CLDN6 has garnered attention as a potential therapeutic target, particularly in adult cancers characterized by its overexpression. However, early data from phase I/II trials have not shown significant improvements when targeting CLDN6 with monoclonal antibodies, nor with CAR-T. Despite of these initial setbacks, CLDNs remain a fertile ground for further research and the development of novel therapeutic interventions.

## Figures and Tables

**Table 1:** Distribution and function of the different claudins in normal tissues.

Type of CLDN	Type of Tissue	Function of CLDN in Normal Tissue
Classic CLDNs		
CLDN 1	Brain, fetal lung alveolar epithelial cells (HFLs), bronchiolar epitheliums, kidney, liver, intestine, pancreatic exocrine cells, and testis [9,30]	Epidermal barrier formation at epidermal thigh junctions
CLDN 2	Pancreatic ducts and exocrine glands [21,31]	Formation of cation channels to promote passage of Na and water
CLDN 3	Thyroid, salivary gland, HFL cells, bronchiolar epitheliums, pancreatic ducts and exocrine glands, liver, colorectal, and kidney [9,31,32,33]	Sealing tight junctions to prevent passage of small ions
CLDN 4	Breast, HFL cells, type II alveolar epitheliums, bronchiolar epitheliums, gastrointestinal mucosa, bile duct, pancreatic ducts and exocrine glands, ovary, prostate, and bladder [32,34]	Paracellular sodium barrier in epithelial cells
CLDN 5	HFL cells, type II alveolar epitheliums, and vascular endothelial cells [32,35,36]	Formation of the blood–brain barrier
CLDN 6	Express in normal tissue in embryo [37,38,39]	Cell-to-cell adhesion
CLDN 7	Bronchiolar epitheliums, pancreatic ducts and exocrine glands, and pancreatic islet endocrine glands [31,32,40]	Paracellular barrier at thigh junctions
CLDN 8	Thyroid, salivary, lung, breast, kidney, adrenal gland, colon, placental, prostate, testis, and skin [9,41]	Paracellular barrier at thigh junctions
CLDN 9	Rarely expressed in normal tissue [42]	
CLDN 10a	Renal tissue [43,44].	Anion channels’ formation
CLDN 10b	Brain, salivary, pancreas, kidney, and adrenal [9,21,43]	Cation channels’ formation
CLDN 14	Renal in thick ascending limb [45,46]	Permeability of Ca in the thick ascending limb of Henle
CLDN 15	Brain, fetal brain, thyroid, heart, lung, stomach, small intestine, pancreas, liver, spleen, kidney, adrenal, uterus, testis, placenta, and skin [9,21,47]	Cation channels’ formation, which decouples the effect of CLDN2
CLDN 17	Brain and kidney [21,48]	Anion channels’ formation
CLDN 19	Eye and kidney [49,50]	Magnesium permeability in thick ascending limb of Henle and eye
Non-classic CLDNs		
CLDN 11	Brain, testis [51]	Permeability of myelin sheaths
CLDN 12	Brain, inner ear, gastrointestinal tract, smooth and striated muscle cells, neurons, and astrocytes [52]	Calcium permeability
CLDN 16	Kidney [49,53]	Role in permeability of Mg in thick ascending limb of Henle
CLDN 18.1	HFL cells and type II alveolar epitheliums [9,54,55,56]	Role in alveolar epithelium maturation and differentiation
CLDN 18.2	Gastric mucosal epithelial cells [57,58]	Role in regulating the formation of gastric mucosal barrier and the diffusion of H+ between the gastric mucosal epithelial cells
CLDN 20	Unknown. Probable no expression in normal tissue	
CLDN 23	Luminal surface of intestinal epithelial cells [18]	

**Table 2:** Expression of the different claudins in various types of cancers and their clinicopathologic correlations.

CLDN	Malignancy	Expression	Clinicopathologic Correlations
CLDN1	Laryngeal SCC [59]	Increased	
	Thyroid [60]	Increased	Higher expressions were noted in PTC > FV-PTC > FTC > FA.
	Esophageal SCC [9,61]	Decreased	Decreased expression is correlated with recurrence status, short DFS, and OS.
	Colorectal [62,63,64,65,66]	Increased	Associated with cancer stemness and chemoresistance. Decreased expression is significantly associated with the progression of histopathologic grade, larger tumor size, vascular invasion, higher pathological tumor stage, high metastatic lymph node ratio, and worse OS and PFS.
	Lung adenocarcinoma [67]	Increased	Overexpression is associated with chemoresistance (to cisplatin and doxorubicin).
	Breast [68,69,70,71]	Increased	High expression is associated with aggressive forms of BC, including inflammatory BC, hereditary BC, some high-grade invasive ductal carcinomas, and the basal-like subtypes. Expression is noted to increase following neoadjuvant chemotherapy.
	BCC of skin [72]	Increased in low-grade BCC	
	Prostate [73]	Increased	Expression is associated with lower pT, low Gleason score, and reduced risk of PSA recurrence.
	Ovarian cancer [9,74]	Increased	
CLDN2	Lung adenocarcinoma [75,76]	Increased	
	Oral SCC [77]	Increased	Increased expression is associated with shorter RFS.
	Breast [78]	Increased	
	Colorectal [63,66,79]	Increased	Increased expression is associated with replacement-type liver metastasis, which carries a worse prognosis compared to the desmoplastic type.
CLDN3	Breast [9,70,78,80,81]	Increased in bilateral breast cancer (BC)	Loss of CLDN3 is more prominent in ER-negative subgroup of bilateral BC. Expression is associated with tumor size > 2 cm and with menopause. Expression is noted to decrease following neoadjuvant chemotherapy.
	Prostate [82,83]	Decreased. However, other study showed increased levels [9]	Decreased expression is associated with CRPC, cases with Gleason score ≥ 8, and locally advanced cases. It is also associated with worse DFS and OS.
	Colorectal	Decreased [84], but other study showed increased expression [66]	CRC is characterized by cytoplasmic expression instead of apical and lateral membrane expression of CLDN3. High expression is associated with worse OS in CMS2 and CMS3 molecular subtypes [85]. Expression is negatively associated with CD4+ T cell infiltration.
	Gastric [86]		CLDN3 has high expression in the immunologically cold tumors and negative correlation with CD8+ T cells in GC.
	Ovarian [9,74]	Increased	Overexpression is correlated with worse OS and PFS.
	Lung [9]	Increased	
CLDN4	Breast [9,78,80,87,88,89,90,91,92]	Increased (more in unilateral BC compared to bilateral BC)	Increased expression is associated with poor OS and with a higher level of circulating tumor DNA. Its expression has a negative correlation with ER and PR and a positive correlation with HER2-neu. CLDN-low TNBC phenotypically behaves like mammary stem cells or epithelial precursor cells and has a poor prognosis associated with early onset of cancer, high histology grade, large tumor size, lymphocytic infiltration, and low local recurrence rate.
	BCC of skin	Decreased in low-grade BCC	
	Oral SCC [77]	Decreased	Reduced expression had a negative impact on RFS.
	Lung [9]	Increased	
	Ovarian [9,74,93]	Increased	CLDN4 can be used to differentiate serous carcinomas (expressed in all cases) from peritoneal mesothelioma (not expressed). Overexpression is correlated with worse OS and PFS.
	Cervical [94]	Increased	Overexpression promotes cervical cancer cell migration and invasion.
	Endometrial [95]	Decreased or absent but increased in uterine carcinosarcomas [96]	
	Prostate [97]	Increased at both RNA and protein levels	Expression is more pronounced in lower-grade primary tumors and also in metastases.
	Pancreatic [9]	Increased	
	Gastric [9,98]	Increased	Higher expression is noted in intestinal rather than diffuse type.
	Colorectal [66]	Increased	
CLDN5	Breast [78]	Decreased	Increased expression is associated with better OS.
	Oral SCC [77]	Decreased	
	Ovarian [74]	Decreased	
	Cervical [99]	Decreased	
	Colorectal [63,66]	Decreased	
CLDN6	Breast [78,100,101]	Increased in ERβ+	Decreases the invasion and migration of breast cancer cells.
	Endometrial [102]	Increased	Aberrant CLDN6 expression promotes tumor growth and invasion in endometrial cancer tissues.
	Cervical [103]	Increased	Overexpression is associated with lymph node metastasis and lymphovascular infiltration. It also contributes to chemoresistance.
	Colon [104]		Expression is associated with decreased migration and invasion abilities of cells.
	Gastric [105,106,107]	Increased	It enhances an array of proteins important for the EMT. Its expression is linked to worse OS in intestinal-type gastric cancer.
	Ovarian [74]	Increased	Overexpression is correlated with worse OS and PFS.
CLDN7	Breast [78,81,90,91,92]	Increased in poorly differentiated tumors.	CLDN-low TNBC phenotypically behaves like mammary stem cells or epithelial precursor cells and has a poor prognosis associated with early onset of cancer, high histology grade, large tumor size, lymphocytic infiltration, and low local recurrence rate.
	Oral SCC [77]	Decreased	Reduced expression is associated with the tumor stage and presence of lymph node metastases.
	Ovarian [9,74]	Increased	
	Colorectal [66]	Increased	
	Esophageal SCC [108,109]	Increased	Reduced expression is associated with the depth of invasion, stage, lymphatics, and lymph node invasion.
	Salivary adenoid cystic carcinoma [110]	Increased	
	Lung [9,111,112]	Increased	Reduced expression is associated with worse OS.
	Thyroid [9]	Increased	
	Gastric [9,113]	Increased	
	Pancreatic [9]	Increased	
CLDN8	Breast [78]	Decreased	
	Colorectal [63,79]	Decreased	It is highly expressed in desmoplastic metastases.
CLDN9	Breast [78]	Increased	
	Endometrial [42]	Increased	Higher expression is associated with lower 5-year disease-specific OS.
	Ovarian [74]	Increased	
CLDN10	Breast [78]	Decreased	
	Ovarian [74]	Increased	Overexpression is correlated with good OS, PFS, and post-progression survival.
CLDN11	Breast [78]	Decreased	Increased expression is associated with better OS.
	Ovarian [74]	Decreased	
	Gastric [114]	Decreased	Significantly associated with smoking, alcohol, Helicobacter pylori infection, and Borrmann classification.
	Colorectal [66]	Decreased	Overexpression is associated with worse OS. There is a positive correlation with macrophage, dendritic cell, and CD4+ T cell infiltration.
CLDN12	Cervical [115]	Increased. It is expressed throughout the cytoplasm of both LSIL and HSIL, with variable signal intensity in SCC	Reduced expression is associated with worse DFS and RFS.
	Colorectal [66]	Increased	Expression is positively correlated with CD4+ T cell infiltration.
CLDN14	Breast [78]	Increased	Increased expression is associated with poor OS.
	Gastric [116]	Increased	
CLDN15	Breast [78]	Decreased	
	Ovarian [74]	Decreased	Overexpression is predictive of a good prognosis.
CLDN16	Ovarian [74]	Increased	Overexpression is correlated with worse OS and PFS.
	Breast [117]	Increased	
CLDN17	Hepatocellular carcinoma [118]	Increased	Expression is associated with a poor prognosis.
	Gastric [116]	Decreased	
	Oral cancer [119]	Decreased	Lower CLDN17 expression is associated with higher tumor staging, poorer tumor histological grading, and a worse clinical prognosis.
CLDN18.2	Gastric [120,121,122,123,124,125]	Decreased	Therapeutic targeting available. Negative CLDN18.2 expression and tumor infiltration with CD4+ T-cells or CD8+ T-cells are associated with a better prognosis. Similarly, higher expression is associated with an advanced cancer stage, poor prognosis, and heightened infiltration of CAFs. On the other hand, other studies showed that overexpression is associated with a younger age, a lower invasion depth limited to the mucosa/submucosa, and less frequent lymphovascular invasion. CLDN18.2 expression is usually more positive in the intestinal type compared to the diffuse type, but, in other reports, it was associated with the diffuse subtype. There is no OS difference between patients with and without CLDN18.2 expression.
	Esophagogastric [126]		Triple positivity for Annexin A10, CLDN18, and SOX2 is more frequent in esophagogastric tumors than in other gastrointestinal tract tumors.
	Pancreatic [127,128]	Increased	Expression correlates with lymph node metastasis, distant metastasis, neural invasion, and stage. Nevertheless, higher expression correlates with better OS.
CLDN19	Breast [78]	Decreased	
CLDN20	Breast [78,129]	Decreased	Increased expression is associated with poor OS.
CLDN23	Colorectal [63,66,130]	Decreased	Associated with longer OS in the CMS4/C4 molecular subtypes but shorter OS in the CMS2/C1 subtypes.
	Pancreatic [131]		Has a role in pancreatic cancer cells’ dissociation and hence metastasis.
	Gastric [114]	Decreased	Significantly associated with vessel cancer embolus.

BC: breast cancer; BCC: basal cell carcinoma; CMS2: epithelial and canonical subtype of colon cancer; CMS4: mesenchymal subtype of colon cancer; CRC: colorectal cancer; DFS: disease-free survival; EMT: epithelial–mesenchymal transition; ER: estrogen receptor; FA: follicular adenoma; FTC: follicular thyroid cancer; FV-PTC: follicular variant-papillary thyroid cancer; GC: gastric cancer; LXRβ: liver X receptor β; OS: overall survival; PFS: progression-free survival; PR: progesterone receptor; PTC: papillary thyroid cancer; PSA: prostate-specific antigen; RFS: relapse-free survival; SCC: squamous cell carcinoma; SOX2: (sex-determining region Y)-box 2; TNBC: triple-negative breast cancer.

**Table 3:** Clinical trials targeting various claudins.

Clinical Trial ID	Phase	Drug	Target	Cancer	Results
MONO trial NCT01197885 [12]	IIa	zolbetuximab as a single agent	CLDN18.2	Advanced relapsed or refractory G/GEJ or esophageal adenocarcinomas	PR = 9%
FAST trial NCT01630083 [189]	II	epirubicin + oxaliplatin + capecitabine (EOC)+ zolbetuximab vs. EOC + placebo	CLDN18.2	G/GEJ and esophageal adenocarcinomas	DCR 76.2%
NCT03874897 [190]	I	CLDN18.2 CAR-T	CLDN18.2	Previously treated gastrointestinal cancer	ORR 48.6% DCR 73.0%
Lordick et al., NCT01671774 [191]	I	zolbetuximab alone or in combination with ZA or with ZA plus IL-2	CLDN18.2	Relapsed or refractory G/GEJ or esophageal adenocarcinomas	PFS of 37.3 weeks with zolbetuximab alone vs. 7.1 to 12.7 weeks in other Rx arms. OS of 60.9 weeks in zolbetuximab + ZA + IL-2 arm, numerically higher than other arms
ILUSTRO trial [192]	II	zolbetuximab monotherapy (in ≥third line) vs. zolbetuximab + mFOLFOX6 (in first line) vs. zolbetuximab + pembrolizumab (in ≥third line)	CLDN18.2	Advanced /metastatic G/GEJ adenocarcinoma	ORR 71.4% in zolbetuximab + mFOLFOX group, but % in the other 2 cohorts
SPOTLIGHT trial NCT03504397 [193]	III	mFOLFOX6 + zolbetuximab vs. mFOLFOX6 + placebo	CLDN18.2	Locally advanced or metastatic HER-2-negative G/GEJ adenocarcinoma	PFS 10.61 vs. 8.67 months in Rx vs. placebo group. HR 0.75, *p* = 0.0066
GLOW trial NCT03653507 [194]	III	CAPOX + zolbetuximab vs. CAPOX + placebo	CLDN18.2	Locally advanced or metastatic HER-2-negative G/GEJ adenocarcinoma	PFS 8.21 vs. 6.80 months in Rx vs. placebo group. HR 0.687, *p* = 0.0007
McDermott et al. [13]	Preclinical trial	humanized anti-CLDN6 monoclonal antibody coupled with monomethyl auristatin E (MMAE) via a cleavable linker	CLDN6	CLDN6 high-expressing cell line and xenograft models	
BNT211-01 trial NCT04503278 [195]	I/II	(CAR)-T with or without a CAR-T cell-amplifying RNA vaccine (CARVac)	CLDN6	Relapsed/refractory CLDN6-positive solid tumors	Unconfirmed ORR 33% (57% inpatient with germ cell tumors)
Adra et al. NCT03760081 [196]	II	ASP1650: a chimeric-mouse/human-IgG1 antibody	CLDN6	Testicular germ cell tumors with average of 3 prior lines of therapy	The study was stopped at the end of Simon Stage-I due to a lack of efficacy

ORR: overall response rate; DCR: disease control rate; G/GEJ: gastric/gastroesophageal junction; PFS: progression-free survival; FOLFOX: 5-fluorouracil + folic acid + oxaliplatin; CAPOX: capecitabine + oxaliplatin; PR: partial response; ZA: zoledronic acid.

## Data Availability

No new data were created or analyzed in this study. Data sharing is not applicable to this article.
